# Segmental Acupuncture for Prevention of Recurrent Urinary Tract Infections. A Randomised Clinical Trial

**DOI:** 10.1007/s00192-024-05872-7

**Published:** 2024-07-25

**Authors:** Thomas Ots, Daniela Gold, Paul Ziller, Thomas Kuenzer, Orietta Dalpiaz, Lejla Pesto, Gerda Trutnovsky

**Affiliations:** 1 Department of Anesthesiology and Intensive Care Medicine, Medical University of Graz, Graz, Austria; 2 Private Acupuncture Clinics, Graz, Austria; 3 Department of Obstetrics and Gynecology, Medical University of Graz, Auenbruggerplatz 14, 8036 Graz, Austria; 4 Institute of Medical Informatics, Statistics and Documentation, Medical University of Graz, Graz, Austria; 5 Department of Urology, Medical University of Graz, Graz, Austria; 6 Present Address: Department of Urology, Hospital of Leoben, Graz, Austria

**Keywords:** Segmental acupuncture, Auriculotherapy, Urinary tract infection, Cranberry

## Abstract

**Introduction and Hypothesis:**

Urinary tract infections (UTIs) are a common medical problem and prophylaxis of recurrent UTIs is an ongoing clinical challenge. In the present study we examined whether acupuncture is able to prevent recurrent UTIs in women.

**Methods:**

This multicentre randomised controlled trial, based at a University clinic and private acupuncture clinics, recruited women suffering from recurrent uncomplicated UTIs. Participants were randomised to the acupuncture group or control group. Acupuncture therapy consisted of 12 treatments over a period of 18 weeks, using a set of predefined body and ear acupuncture points. Cranberry products were recommended to all participants as standard of care.

**Results:**

A total of 137 women were randomised (68 acupuncture, 69 control group) and occurrence of UTIs at 6 and 12 months could be assessed in 123 and 120 women respectively. Acupuncture combined with cranberry slightly increased the proportion of UTI-free women compared with cranberry alone at 6 months (59% vs 46%, *p* = 0.2). Between 6 and 12 months the proportion of UTI-free women was significantly higher in the acupuncture group (66 vs 45%, *p* = 0.03). The number of UTIs decreased from baseline to 12 months in both study groups. The number of UTIs at 12 months was significantly lower in the acupuncture group (median difference 1, *p* = 0.01).

**Conclusions:**

Segmental acupuncture may be an effective treatment option for women with recurrent UTIs over a longer follow-up period and may limit antibiotics use. Further studies are needed.

**Supplementary Information:**

The online version contains supplementary material available at 10.1007/s00192-024-05872-7

## Introduction

Urinary tract infections (UTIs) are considered to be the most common bacterial infections, with approximately 80% of all UTIs occurring in women [1]. Nearly 1 in 3 women is expected to suffer from at least one episode of UTI by the age of 24 years and almost half of all women will experience one UTI during their lifetime [2]. Recurrences affect approximately 20–30% of women with initial UTI, although recurrence rates vary widely [3].

Recurrent UTIs, defined as two UTIs within 6 months or three UTIs within 12 months [4], are a common problem seen in clinical practice with important medical, social and financial implications. Prophylaxis of recurrent UTIs is an ongoing challenge, with several different management strategies being used [5, 6]. Low-dose antibiotic prophylaxis for several months is reported to be effective but should not be administered first line, because it fosters the development of antibiotic resistance of the causative microorganisms, as well as the commensal flora [5, 7–9]. In postmenopausal women vaginal oestrogen therapy reduces symptomatic UTI episodes [1, 10]. Further prevention strategies include the oral immunostimulant OM-89, the vaginal vaccine Urovac, lactobacilli prophylaxis, cranberry products, and acupuncture [10–13]. American cranberries have been used in the prevention of UTIs for many years. The latest Cochrane update supports the use of cranberry products to reduce the risk of symptomatic UTIs in women with recurrent UTIs [14].

The use of acupuncture for UTI treatment has been studied for over 20 years [15–17]. So far, two randomised controlled trials assessed the effect on the prevention of recurrent UTIs with positive results [15, 17]. Acupuncture points in those studies were chosen according to patients´ individual diagnoses following Traditional Chinese Medicine (TCM) patterns [15, 16].

Segmental acupuncture, based on segmental anatomy, is in part an alternative model to the traditional Chinese meridian system. The locations of the needles are determined by the corresponding segments of the affected organs. This allows standardised treatment regimens without in-depth knowledge of TCM. Combination with auriculotherapy has been proven to be useful [18].

The aim of the present study was to assess the effect of segmental acupuncture combined with auriculotherapy in the treatment of recurrent UTIs in women. We hypothesised that women receiving acupuncture treatment in addition to standard treatment were more likely to have no UTIs at 6 months than women with standard treatment only.

## Materials and Methods

This was a multicentre randomised controlled clinical trial with a 12-month follow-up conducted at a University Clinic of Obstetrics and Gynaecology, a University Clinic of Urology, and 7 private acupuncture clinics within Austria. Patients with recurrent uncomplicated UTIs were invited to participate. Inclusion criteria were a history of at least two symptomatic UTIs within the last 6 months or at least three symptomatic UTIs within the last 12 months. UTIs had to be diagnosed by a health care provider with a dip-stick test and at least one positive urine culture within the last year was required. Exclusion criteria were pregnancy, diabetes, an indwelling urine catheter, renal insufficiency, transplantation, or immunodeficiency. Women with pelvic organ prolapse stage ≥ 2 and post-void residuals > 100 ml were excluded. The study was designed according to the Consolidated Standards of Reporting Trials (CONSORT) and STandards for Reporting Interventions in Clinical Trials of Acupuncture (STRICTA) guidelines [19].

Eligible and consenting patients were randomised to acupuncture treatment plus cranberry treatment or cranberry treatment only via the central computerised system “randomiser” at a ratio of 1:1. Blinding of clinical assessors was not feasible, because follow-up assessments were usually done by the same investigator, who randomised patients and informed them about study allocation.

Patients randomised to the acupuncture group received acupuncture treatments at 1 of 7 private acupuncture clinics participating in the study. Women randomised to the control group were asked not to undergo acupuncture treatment for any medical reason within 6 months after being included in the study. All study participants were counselled about urinary symptoms and protective behaviour. Daily use of cranberry products was recommended as standard of care in both study groups. Cranberry products were provided free of charge for 6 months. All participants received a study diary and were advised how to document urinary symptoms and intake of cranberry products.

In the case of UTI symptoms, i.e. frequency or urgency, dysuria, suprapubic tenderness, haematuria, or fever (> 38 °C), study participants were advised to access their health care provider. For diagnosis of UTI a dip-stick test of clean-catch midstream urine and/or a urine culture was recommended. Antibiotics treatment regime was individually determined by health care providers, taking into account information about uropathogens, resistance patterns and adverse effects profiles.

All study participants were scheduled for follow-up visits at 6 and 12 months. During the visits the study diaries were reviewed, amounts of UTIs assessed and questionnaires administered.

### Acupuncture Treatment

All acupuncture treatments were performed by medical doctors with certified acupuncture diplomas of the medical board of Austria and at least 5 years of practice. Physicians were advised to limit conversations with study participants to a minimum. A total of 12 treatments was performed over a period of 18 weeks. Treatment sessions took about 30 min and were timed according to a defined time schedule with increasing time intervals. Patients were placed in a lateral position, which was changed each time to ensure balanced stimulation.

**Fig. 1:**
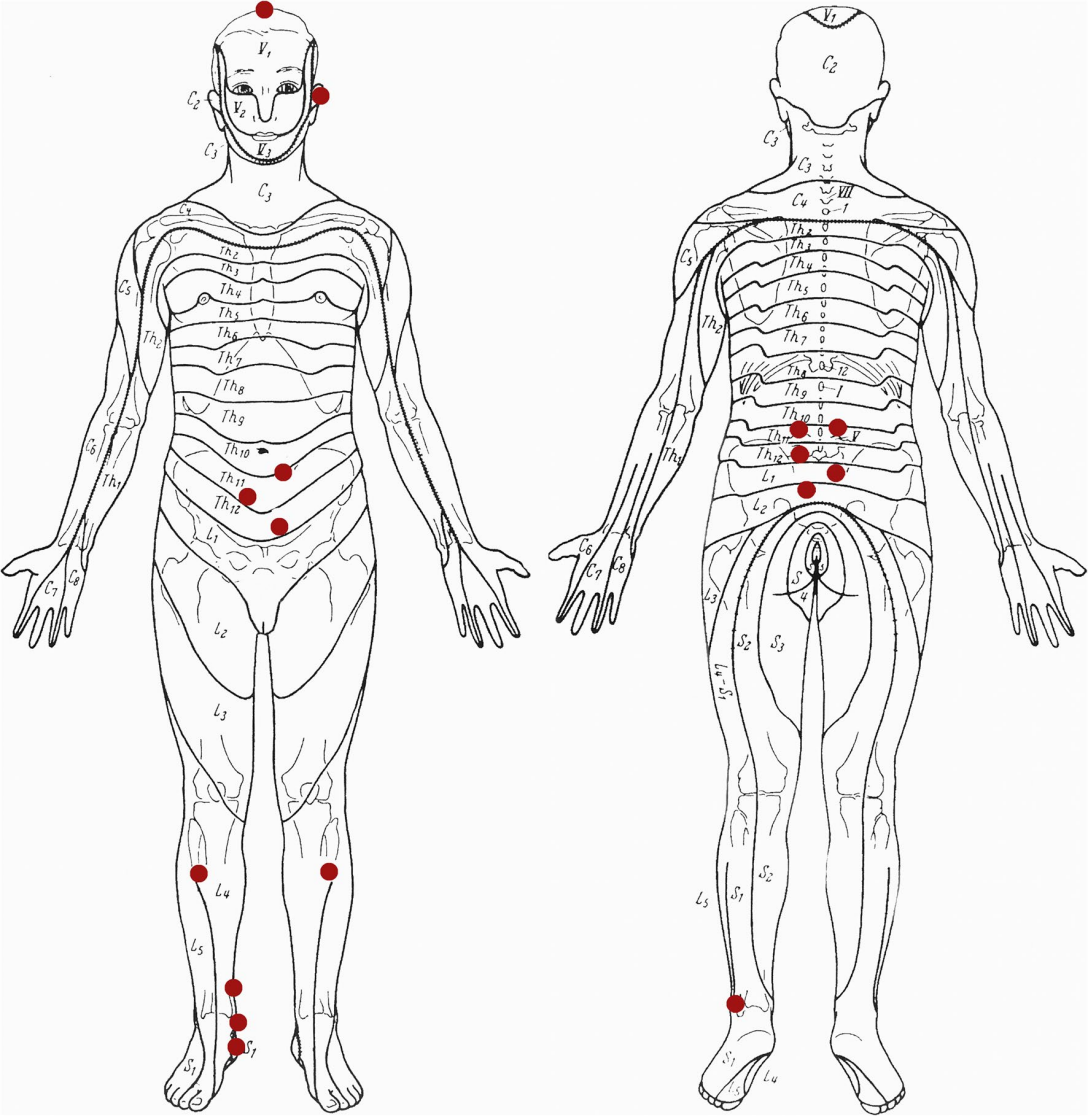
Acupuncture body points and corresponding dermatomes

**Fig. 2:**
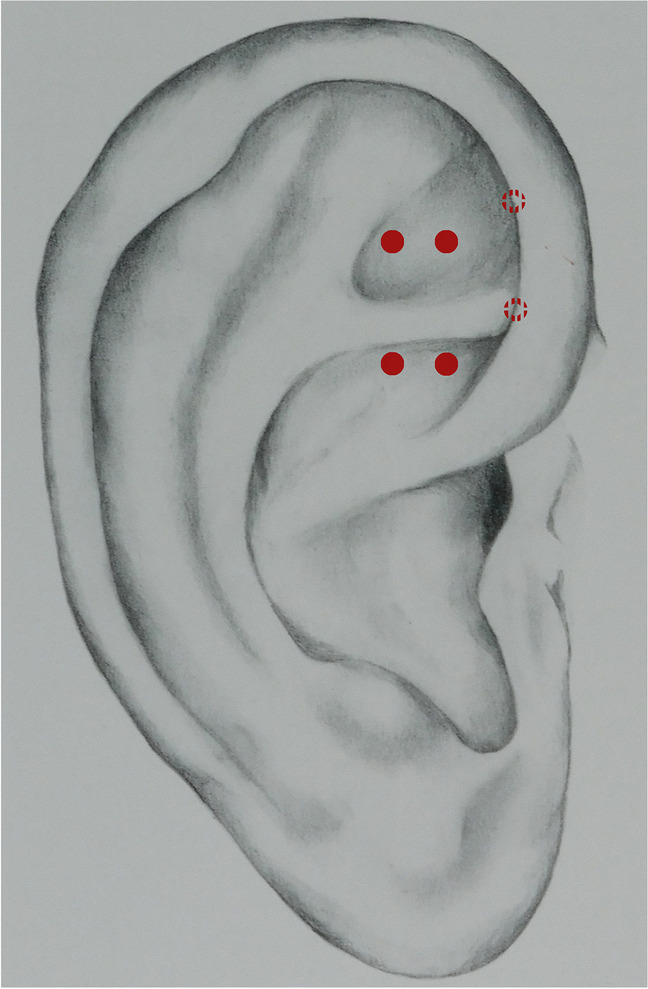
Acupuncture ear points

Acupuncture was performed according to the physiological concept of segmental acupuncture [20, 21], which has been tested and practiced successfully in the clinic of the first author for many years. A predefined set of acupuncture body and ear points (Figs. 1, 2) was used without seeking an individual TCM-based diagnosis.

The organs of the urogenital system are innervated by nerves of the segments Th10 to S4 and local needle stimuli were applied in the area from Th11 to L1. Ventrally, three needles were placed at the lower abdomen in proximity to the acupuncture points Kidney 13 to 15. Dorsally, five needles were placed at the lower back in proximity to Bladder 24 to 28. Needles were placed unilaterally per segment about 1 cm from the midline, except for Bladder 24, which was pricked bilaterally.

These loci were combined with distant points located within the segments L4/L5, S1 and S2 corresponding to the urogenital organs. On the lower limbs the needles were placed approximately at acupuncture points Stomach 36, Spleen 6, Kidney 6, Kidney 7 and Bladder 60. Owing to patients’ lateral position, all points except Stomach 36 were pricked unilaterally. On the head the acupuncture point Du 20, widely used as a calming point, was added.

Acupuncture needles (TEWA® 0,=.3 × 30 mm; asia-med GmbH, Pullach, Germany) were inserted approximately 15 mm under the skin without especially seeking the “deqi sensation” (a sensation described as numbness, heaviness and distension).

For auriculotherapy smaller acupuncture needles (TEWA® 0,2 × 15 mm; asia-med) were inserted up to 1 mm on the following ear points: Kidney, Bladder, Sympathetic, Lower Pelvis, Knee in the middle of the triangular fossa, as described in French acupuncture (Fig. 2). Investigators were instructed to use an electrical potentiometer for the exact localisation of the respective points.

### Cranberry Treatment

The following products were supplied to participants independent of study group allocation: Cranberry Granulate Alpinamed® (Gebro Pharma GmbH, Fieberbrunn, Austria), containing 225 mg proanthocyanidin (PAC), Cranberry capsules Preisel-Caps® (Caesaro Med GmbH, Leonding, Austria) containing 36 mg PAC and Cranberry capsules Urgenin® (Madaus GmbH, Cologne, Germany) also containing 36 mg PAC. Recommended intake was once daily for the Cranberry Granulate and twice to thrice daily for the Cranberry capsules.

### Outcome Measures

The primary study outcome was the proportion of women without UTIs at 6 months. Secondary outcomes were the number of UTIs, antibiotics medication, use of cranberry products, health-related quality of life (HrQoL) and treatment satisfaction at 6 months. To assess the long-term effect of acupuncture we analysed the proportion of UTI-free women and number of UTIs at 12 months as further study outcomes.

The number of UTIs, antibiotics and cranberry use were assessed during review of the study diaries at the follow-up visits.

The HrQoL was assessed using the King’s Health Questionnaire (KHQ) at baseline and at 6 months. The 32-item, condition-specific instrument was validated to assess HrQoL in women with lower urinary tract conditions and has been widely used in research. The German-language version of the KHQ was validated in women with stress urinary Incontinence [22], but may also be used in patients with other bladder problems.

Treatment satisfaction was assessed at 6 months using an adopted German version of the “Client Satisfaction Questionnaire—CSQ8”, a validated tool for measuring global patient satisfaction at the end of treatment [23].

In the case of missing follow-up visits patients were contacted via telephone and information on cranberry intake, urinary symptoms and other missing data was collected.

### Statistical Analysis

Sample-size estimation was performed for the primary outcome of the proportion of UTI-free women at 6 months. Based on the existing literature [15, 16], the difference in the proportion of women free of UTIs between the acupuncture and the control group was expected to be between 25 and 50%. The sample size calculated to achieve a power of 80% was increased by an anticipated drop-out of 10 women per group (17%), resulting in a total of 136 patients planned for study inclusion. Summary statistics for continuous variables are presented as median and quartiles, and for discrete variables as count and proportion. The analysis of the main and secondary outcomes was calculated using Fisher’s exact test for categorical data and the Mann–Whitney *U* test for continuous and count data. Two-sided *p* values < 0.05 were considered statistically significant. Differences between the groups were calculated using the odds ratio (OR) for binary outcomes and using Hodges–Lehmann median of pairwise differences for continuous outcomes. As a sensitivity analysis, we analysed the binary and count data outcomes adjusting for patient age using logistic regression models or quasi-Poisson regression models, summarising the effects as adjusted odds ratios and adjusted incidence rate ratios respectively. Data were analysed using the intention-to-treat principle. The main findings did not change using per-protocol analysis. No imputation for missing data was performed. All data analyses were done using R [24].

The study was approved by the local Ethics Committees and all participants provided written informed consent.

## Results

**Fig. 3:**
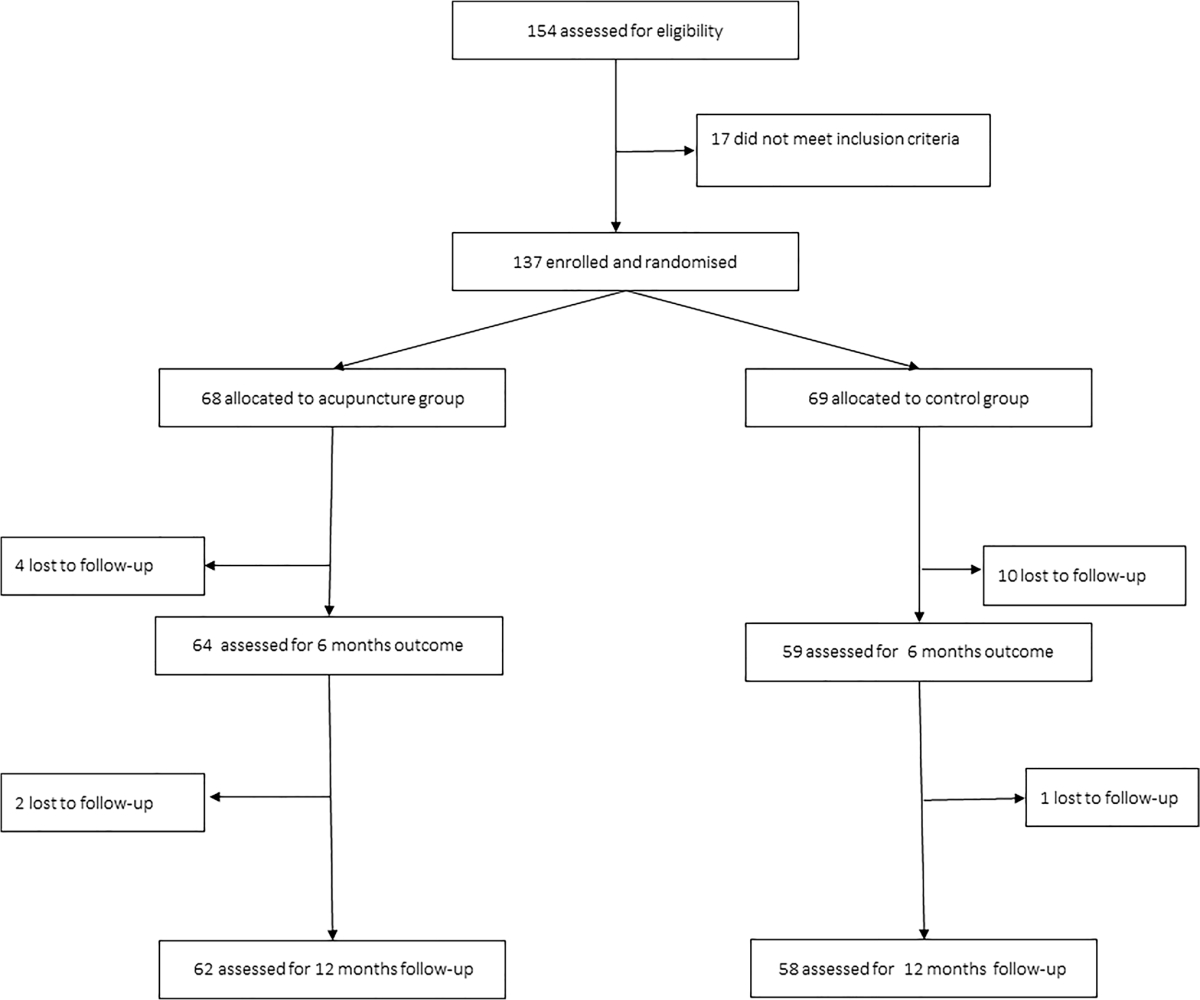
Consolidated Standards of Reporting Trials flow-chart of study participants

Between March 2015 and April 2021 a total of 137 patients were enrolled. Sixty-eight women were allocated to acupuncture treatment and 69 women to the control group (Fig. 3). Patient characteristics of the two groups of the study population were comparable (Table 1).

**Table 1:** Baseline characteristics of the study population

	Acupuncture group (*n* = 68)	Control group (*n* = 69)
Age (years)	35 (27—52)	42 (27–61)
UTIs during the last 12 months (reported)	4 (3—6)	5 (3–6)
UTIs during the last 12 months (documented)	3 (0–4)	3 (0–4.3)
Number of antibiotic treatments during the last 12 months	4 (3–5)	4 (3–6)
Parity		
0	38 (57)	33 (49)
1	7 (10)	14 (21)
2	15 (22)	14 (21)
3 or more	7 (10)	7 (10)
Current hormonal contraception	13 (19)	13 (19)
Postmenopause	19 (28)	29 (43)
Current local hormonal replacement therapy	10 (15)	10 (15)
Current sexual activity	48 (73)	52 (76)
UTIs after sexual activity	30 (47)	27 (40)
Current or past smoking	13 (19)	14 (21)
Comorbidities		
Recurrent vaginal infections	34 (51)	31 (46)
Urinary incontinence	13 (19)	8 (12)
Previous hysterectomy	8 (12)	11 (16)
Previous malignancy	4 (6.1)	4 (5.9)
Previous acupuncture treatments	39 (58)	34 (50)
Previous treatment satisfaction		
Very satisfied or satisfied	27 (71)	24 (73)
Moderate satisfied	7 (18)	4 (12)
Little or no satisfaction	4 (10)	5 (15)

Data are presented as median (interquartile range) or *n* (%)

**Fig. 4:**
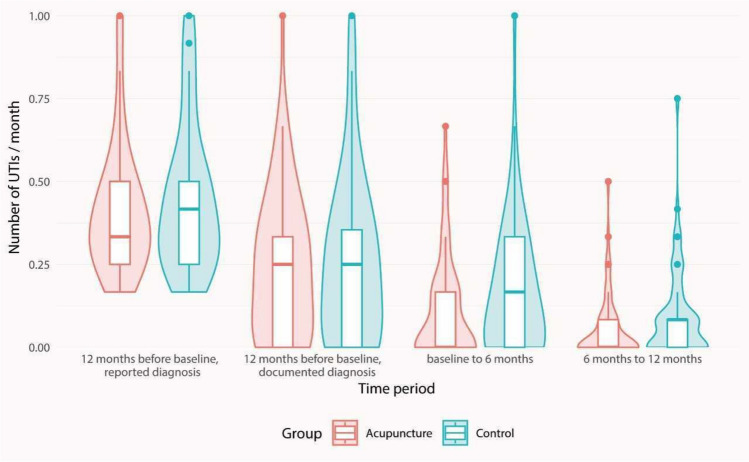
Median number of urinary tract infections (UTIs) per month at study inclusion (baseline), at 6 months’ and at 12 months’ follow-up. Reported diagnosis: number of UTIs reported by participants. Documented diagnosis: number of reported UTIs confirmed by a health care provider (diagnosed using the dip-stick test)

The primary endpoint, occurrence of UTIs at 6 months, could be assessed in 123 patients (64 in the acupuncture group, 59 in the control group). Drop-outs were more common in the control group and among younger patients, with a median age of the drop-outs of 34.0 years in both groups. In the acupuncture group, the proportion of UTI-free patients at 6 months was 59% compared with 46% in the control group (OR 1.72; 95% CI 0.80–3.77; *p* = 0.2). The difference between study groups increased at 12 months’ follow-up, with significantly more patients being UTI free between 6 and 12 months in the acupuncture group (66% vs 45%, OR 2.38; 95% CI 1.08–5.37; *p* = 0.03), as shown in Table 2. In comparison with the 12 months preceding study inclusion, the rate of UTIs decreased markedly in both study groups at both 6 months’ and 12 months’ follow-up (Fig. 4). The number of UTIs at 12 months was significantly lower in the acupuncture group than in the control group (median difference 1, *p* = 0.01). Owing to the unequal drop-out rates and the resulting unforeseen age imbalance between the groups, we additionally conducted sensitivity analysis by adjusting for patient age using logistic regression models or quasi-Poisson regression models. Adjusting for this important covariate did not qualitatively change the main findings.

**Table 2:** Study outcomes at 6 and 12 months

	Acupuncture group, *n* = 64/ *n* = 62^a^	Control group, *n* = 59/*n* = 58^a^	Odds ratio (95% CI)	*p* value*
Primary outcome				
Patients with no UTIs at 6 months	38 (59)	27 (46)	1.72 (0.80–3.77)	0.2
Secondary outcomes at 6 months				
Number of UTIs during the last 6 months	0 (0–1)	1 (0–2)		0.08
Patients with antibiotics treatment during the last 6 months	14 (22)	22 (37)	0.47 (0.20–1.12)	0.08
Secondary outcomes at 12 months				
Patients with no UTIs during the last 12 months	29 (47)	15 (26)	2.50 (1.09–5.90)	0.02
Number of UTIs during the last 12 months	1 (0–2)	1 (0.25–3)		0.01
Patients with no UTI between 6 and 12 months	41 (66)	26 (45)	2.38 (1.08–5.37)	0.03
Number of UTIs between 6 and 12 months	0 (0–1)	1 (0–1)		0.02
Antibiotics treatment between 6 and 12 months	9 (14)	15 (27)	0.46 (0.16–1.25)	0.1

Data are presented as median (interquartile range) or *n* (%)

UTIs were diagnosed by health care providers using a dip-stick test

*UTI* urinary tract infection

*p values derived from Fisher's exact tests and Mann–Whitney *U* test respectively

^a^Numbers represent groups at 6 months and at 12 months

The percentage of women requiring antibiotics treatment decreased noticeably from pretreatment (97% vs 99%) to 6 months (22% vs 37%) and to the time period between 6 and 12 months (14% vs 27%) in the acupuncture group and the control group respectively. The study groups did not differ in terms of types of antibiotics treatment regimens received, which consisted of fosfomycin (19%), nitrofurantoin (13%), trimethoprim (10%), cefuroxime (8%) or other antibiotics (6%) or were unknown (44%).

Fifty-six patients (93%) randomised to acupuncture received all 12 treatments, 4 patients (6%) received between 9 and 11 treatments and in 8 patients the actual number of treatments was not recorded. In the control group, 1 woman received acupuncture treatments during the study period. The majority of patients in both study groups used cranberry products on a regular daily basis during the 6-month study period (median frequency 1.0 (0.9–1.0) vs 1.0 (0.75–1.0), *p* = 0.6). According to the study diaries and follow-up assessments 67% used Alpinamed ®Cranberry Granulate, 13% Preisel-Caps® tablets and 20% Urgenin® tablets, up to once (59%), twice (37%) or three times daily (4%). During days with urinary symptoms patients changed cranberry intake to once (20%), twice (59%) and three times or more daily (20%).

Treatment satisfaction at 6 months could be assessed in 122 women (89%). Overall treatment satisfaction was significantly higher in the acupuncture group (median CSQ8 sum score 32 vs 29, *p* < 0.001), with 88% being very satisfied and 77% judging their treatment as being very helpful for their urinary symptoms, compared with 55% and 57% in the control group respectively. The KHQ was completed by 127 (93%) at baseline and 75 (55%) at 6 months’ follow-up, with no significant differences in subscales between study groups.

No adverse events were observed or reported during the entire study period.

## Discussion

This was the first study to analyse the efficacy of segmental acupuncture in combination with auriculotherapy in women with recurrent UTIs in the course of a randomised controlled trial with 12 months’ follow-up. We found that acupuncture combined with regular cranberry intake in women with recurrent UTIs reduced the risk of subsequent UTIs, compared with cranberry intake only. Although the primary outcome, i.e. the proportion of UTI-free women at 6 months, did not show a statistically significant difference between the groups, the secondary study outcomes provided important clinical findings. At 12 months the proportion of UTI-free women was significantly higher in the acupuncture group than in the control group, suggesting a long-term benefit of acupuncture. Acupuncture was well accepted, with 93% of participants attending all treatment sessions and no adverse events being recorded.

The study was designed according to the CONSORT and STRICTA guidelines and was performed with a high standard of conduct from randomisation to acupuncture treatment, data management and statistical analysis. Objective outcomes, i.e. number of UTIs and antibiotics use, were combined with subjective outcomes, i.e. HrQoL and treatment satisfaction. The use of cranberry products, the most commonly used self-medication, was monitored with the use of study diaries. Although three different cranberry products were provided, daily total PAC doses were comparable. The equivalent and regular intake in both study groups provided additional information regarding the effectiveness of regular PAC, although this was not a predefined study outcome.

Our results are in line with the findings of the two previous RCTs studying the effect of acupuncture on the prevention of recurrent UTIs. In the study by Alraek et al. 73% of women in the acupuncture group were free of UTIs at 6 months compared with 52% of women in the control group with no treatment [16]. Positive results were also reported by Aune et al., with 85% of women being free of UTI at 6 months in the acupuncture group, compared with 58% in the sham group and 36% in the control group [15].

In both Norwegian studies [15, 16], acupuncture points were chosen according to the patients’ individual TCM diagnosis with exact localisation of points and attention to “deqi” needling. In our study we used segmental acupuncture, which can be applied according to standardised treatment protocols and can be learned und used easily without acquiring deeper knowledge of TCM. Segmental acupuncture is based on the segmental anatomy presented by Henry Head in 1894. The human being as a vertebral being is arranged metamerically, i.e. the spinal cord of each vertebra corresponds to a specific segment. All segments show an identical structure: a spinal nerve (neurotome) runs from the spinal cord peripherally to the skin (dermatome) and has branches to muscles (myotome), bones (sclerotome), organs (viscerotome) and the vegetative nervous system (sympathetic nerve). It is assumed that the acupuncture stimuli proceed segmentally and follow the traditional TCM meridians only to a certain degree [21]. This neurophysiological explanation of the effects of acupuncture has become increasingly recognised in Chinese and Western literature [25, 26]. The long-term effect of acupuncture may be explained in terms of an immune modulating effect [17, 20]. We did not use sham acupuncture in our control group, because sham needling on overlapping dermatomes has been shown to produce clinically relevant effects [27, 28].

In our study cranberry products were provided free of costs for the first 6 months and monitored with study diaries in all study participants in order to increase compliance and minimise uncontrolled self-medication. According to the patient’s diaries cranberry use was sustained and very regular and possibly higher than in a general population after medical recommendation. This may explain the good treatment result in the control group (cranberry use only). Cranberries contain two compounds with anti-adherence properties, the so-called *Vaccinium macrocarpon* and proanthocyanidins components. These components are meant to prevent fimbriated *Escherichia coli* from adhering to uroepithelial cells by inhibiting the synthesis of P fimbriae and by deforming the cell body of the bacterium [29]. Our results are in line with the latest Cochrane review update, which found that the use of cranberry products reduces the risk of symptomatic, culture-verified UTIs in women with recurrent UTIs [14].

Several study limitations need to be considered. It is possible that the increased interaction that the acupuncture group received may have influenced the study outcome. Although we tried to minimise conversations during acupuncture sessions, we cannot exclude that some additional counselling occurred. Although drop-outs were anticipated, the percentage of participants lost to follow-up was higher in the control group (16%) than in the acupuncture group (6%). Drop-outs were younger than the overall study sample, which must be considered in the interpretation of this study as a possible source of attrition bias. However, unplanned sensitivity analyses indicate that the results do not change even after adjustment for patient age. The lack of blinding may have influenced the higher rate of reported urinary symptoms and the higher drop-out rate in the control group than in the acupuncture group. Some patients did not attend their follow-up visits, but were contacted via telephone at a later stage, and limited recall may have occurred. KHQ data were partly incomplete and interpretation is therefore limited. Furthermore, the positive finding favouring segmental acupuncture in the secondary outcomes should be interpreted as an explorative result.

In conclusion, acupuncture may be an effective treatment option for women with recurrent UTIs over a longer follow-up period. Further studies will need to investigate the use of segmental acupuncture, with and without auriculotherapy, versus cranberry intake, and clarify the therapeutic role of acupuncture and the minimum amount of acupuncture sessions needed. Future investigation may also include a sham controlled trial. The current results are promising for further integration of acupuncture into conventional medicine.

## Supplementary Information

Below is the link to the electronic supplementary material.

Supplementary file1 (PDF 244 KB)

## Data Availability

Study data are available on request from the corresponding author.
